# Histones join the fight against bacteria inside cells

**DOI:** 10.7554/eLife.00302

**Published:** 2012-11-13

**Authors:** Roberto Kolter

**Affiliations:** **Roberto Kolter** is an *eLife reviewing editor*, and at Harvard Medical School, Boston, United Statesroberto_kolter@hms.harvard.edu

**Keywords:** innate immunity, histones, lipid droplet, anti-bacterial, B. subtilis, D. melanogaster, E. coli, Mouse

## Abstract

Experiments on *Drosophila* have shown that the histones that are normally bound to lipid droplets inside cells can be released to provide protection against infection.

**Related research article** Anand P, Cermelli S, Li Z, Kassan A, Bosch M, Sigua R, Huang L, Ouellette AJ, Pol A, Welte MA, Gross SP. 2012. A novel role for lipid droplets in the organismal antibacterial response. *eLife* **1**:e00003. doi: 10.7554/eLife.00003

**Image** Bacteria multiply in the absence of histones bound to lipid droplets

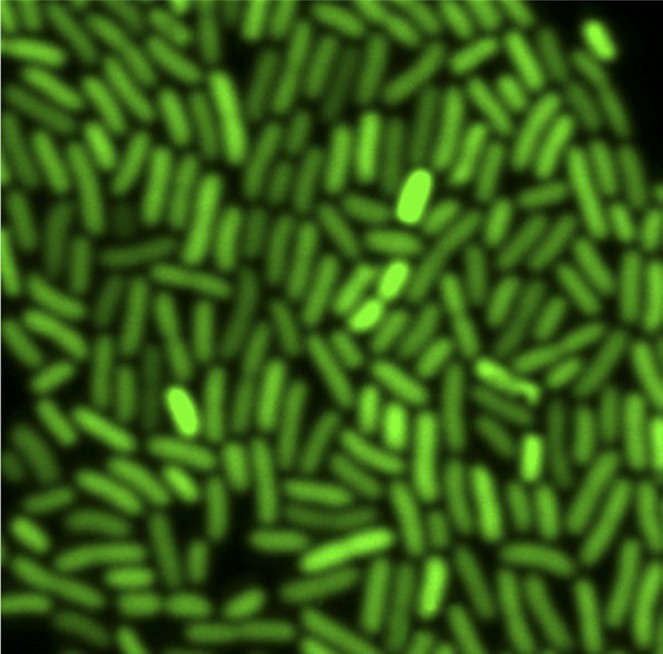

All the plants and animals on our planet have evolved among a myriad of microorganisms and, by and large, the relationships between these microbes and their much larger neighbours have been beneficial. At the same time, microbes have developed many different ways of digesting organic matter and they are always ready to make lunch out of almost any part of any animal or plant that is available. The larger organisms have, therefore, had to evolve immunity mechanisms to cope with the pervasive presence of microbes.

The richness and diversity of these immunity mechanisms is striking. Some immunity is adaptive, and is tailored to deal with the presence of specific microbes, such as the production of highly specific antibodies that help neutralize foreign invaders. Other forms of immunity are innate and offer generic protection against pathogens: our skin, for example, forms a physical boundary that keeps microbes away from places where they should not be. The process of inflammation in tissues is also part of our innate immune system, as are white blood cells. Now, in *eLife*, Steven Gross and co-workers at the University of California at Irvine, the University of Southern California, the IDIBAPS in Barcelona and the University of Rochester describe a new type of innate immunity involving intracellular fat deposits and histones that could well be at work throughout the animal kingdom (Anand et al., 2012).

All living cells contain organelles called lipid droplets that are used to store fat, which is a source of energy for cells, and for years it was thought that this was the sole purpose of these droplets (Walther and Farese, 2012). Now we know that lipid droplets perform many other functions in cells, including the sequestration of histones and other proteins (Beller et al., 2006). This was initially quite surprising because histones are normally bound to DNA to constitute chromatin. However, histones can be quite toxic on their own so it makes sense for any surplus histones to be sequestered on lipid droplets. Indeed, it was shown a decade ago that histone toxicity can defend animal cells against bacteria living outside the cells (Cho et al., 2002).

Given that histones can offer protection against extracellular bacteria, Gross and co-workers—including Preetha Anand and Silvia Cermelli of UC Irvine as joint first authors—decided to ask the following question: might the potential toxicity of the surplus histones stored on lipid droplets be exploited to protect against bacteria ‘inside’ the cell? Working with early-stage embryos of the fruit fly *Drosophila melanogaster*, which contain large numbers of lipid droplets (Figure 1), Anand, Cermelli and co-workers demonstrated that the answer to this question was a resounding yes. Quite strikingly, when the *Drosophila* cells were exposed to various components from the cell walls of a range of bacteria, histones were released from the lipid droplets and demonstrated antibacterial activity.

**Figure 1.**
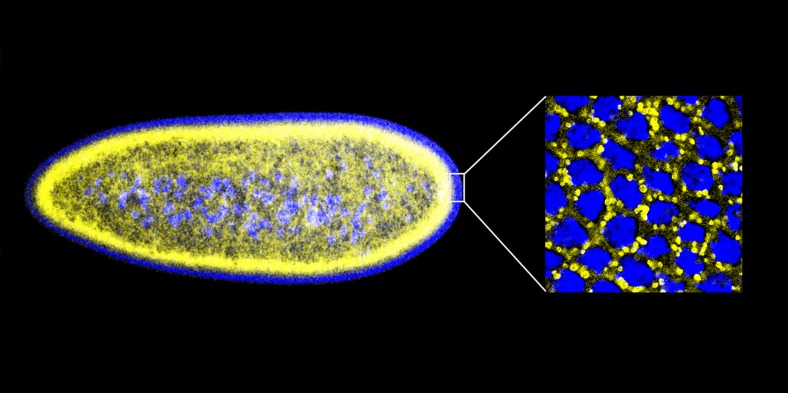
An early *Drosophila* embryo visualized using fluorescence microscopy. Nuclei have been false coloured blue and lipid droplets yellow. This embryo, which is ∼0.5 mm in length, is still a single cell containing many nuclei (a syncytium), and most of these are found on the surface. The detail on the right is an expanded head-on view of the surface inside the white rectangle and shows about 32 densely packed nuclei inside an area of about 25 × 25 µm.

IMAGES: MICHAEL WELTE.

This immediately raised another question: do cells actually rely on histones to protect them against bacteria? One way to explore this further would be to compare the response of cells with and without histones to the presence of bacteria. However, histones are essential for the survival of the host organism, so it is simply not possible to isolate flies that do not contain histones. Luckily, there was another way to proceed because a group led by Michael Welte at Rochester, one of the authors of the present study, had just identified a protein that appeared to be the histone receptor on the lipid droplets (Li et al., 2012). Mutant flies lacking this protein appeared largely normal, but they had irregularly shaped lipid droplets and, crucially, histones could not bind to these droplets. There is a tradition of giving *Drosophila* genes unusual names—the Toll and Spätzle genes, for example, are named after the German words for ‘great’ and ‘egg pasta’ (Weissmann, 2010)—and since the blob-like lipid droplets in flies that did not contain the receptor reminded Welte and co-workers of Jabba the Hutt from ‘Star Wars’, the histone receptor was named *Jabba*.

Anand, Cermelli and co-workers showed that flies without the *Jabba* receptor (*Jabba* mutants) were more sensitive to being killed by several species of bacteria than wild-type flies (whose lipid droplets did contain histones). This increased killing was observed both for developing embryos and adult flies. The researchers went on to obtain provocative preliminary results that histones in lipid droplets in mammalian cells (in this case mice) may also have a role in innate immunity. Therefore, it now appears that lipid droplets, once considered to be simply fat deposits, could in fact constitute part of the immune system of animals by virtue of the fact that they contain histones that may be released upon exposure to bacteria.

Understanding the mechanisms involved in the release of the histones is an exciting challenge for workers in the field. Establishing whether there is direct contact between the bacterial cell wall components and the histones on the lipid droplets will be first step in this process. Importantly, the way the histones kill bacteria still needs to be investigated. One possibility is that the histones released from the lipid droplets adopt a conformational state that renders them able to non-specifically disrupt cells: the key to immunity with self-toxicity, therefore, would be the ability to only adopt this conformational state in the right place at the right time—when bacteria are in the vicinity.

Does this mean that increased fat accumulation within cells leads to an increased immunity against bacterial infection? Not likely—it is the histones in the cell that kill the bacteria, and there should be plenty of those even in lean individuals, so there is no reason to indulge in extra donuts to supplement your lipid droplet supply.
